# Supplementary material for: Heat adaptation of phage T7 under an extended genetic code
Source: Virus Evol. 2021 Dec 1;7(2):veab100. doi: 10.1093/ve/veab100 (PMC8923235; doi:10.1093/ve/veab100)
Supplement: veab100_Supp [file veab100_supp.zip › Sequencing_Analysis.zip!/Sequencing_Analysis/S1/evidence/JC_47.html]

BRESEQ :: Evidence


| New junction evidence | | | | | | | | | | | |
| --- | --- | --- | --- | --- | --- | --- | --- | --- | --- | --- | --- |
|  | | seq id | position | reads (cov) | reads (cov) | score | skew | freq | annotation | gene | product |
| \* | ? | NC\_001604.1 | = 1256 | 0 (0.000) | 154 (0.040) | 67/574 | 22.6 | 99.7% | intergenic (–/–) | *–/–* | –/– |
|  | ? | NC\_001604.1 | 2737 = | 1 (0.000) |  |  |  |  | intergenic (–/–) | *–/–* | –/– |
| Rejected: Coverage evenness skew score above cutoff. | | | | | | | | | | | |

Only 100 of 179 total aligned reads displayed.  

`CGTTTTCGACCACGCTTACGAAATGCTGAAAGAAAACATCCGTTATGATGACATCCGTGACACTGATGACCTGCACGATGCTATTCACATGGCTGCCGATAATGCAGTTCCGCACTACTACGCTGACATCTTTAGCGTAATGGCAAGTGAGGGCATTGACCTTGAGTTCGAAGACTCTGGTCTGATGCCTGACACCAAGGACGTAATCCGCATCCTGCAAGCGCGTATCTATGAGCAATTAACGATTGACCTCTGGGAAGACGCAGAAGACTTGCTCAATGAATACTTGGAGGAAGTCGAGGA‑‑‑‑‑‑‑‑‑‑‑‑‑‑‑‑‑‑‑‑‑‑‑‑‑‑‑‑‑‑‑‑‑‑‑‑‑‑‑‑‑‑‑‑‑‑‑‑‑‑‑‑‑‑‑‑‑‑‑‑‑‑‑‑‑‑‑‑‑‑‑‑‑‑‑‑‑‑‑‑‑‑‑‑‑‑‑‑‑‑‑‑‑‑‑‑‑‑‑‑‑‑‑‑‑‑‑‑‑‑‑‑‑‑‑‑‑‑‑‑‑‑‑‑‑‑‑‑‑‑‑‑‑‑‑‑‑‑‑‑‑‑‑‑‑‑‑‑‑‑‑‑‑‑‑‑‑‑‑‑‑‑‑‑‑‑‑‑‑‑‑‑‑‑‑‑‑‑‑‑‑‑‑‑‑‑‑‑‑‑‑‑‑‑‑‑‑‑‑‑‑‑‑‑‑‑‑‑‑‑‑‑‑‑‑‑‑‑‑‑‑‑‑‑‑‑‑‑‑‑‑‑‑‑‑‑‑‑‑‑‑‑‑‑‑‑‑‑‑‑‑‑‑‑‑‑‑‑‑‑‑‑‑‑‑‑‑‑‑‑‑‑‑‑‑‑‑‑‑‑‑‑‑‑‑‑‑‑‑‑‑‑‑  >  NC_001604.1/954‑1256`  
`‑‑‑‑‑‑‑‑‑‑‑‑‑‑‑‑‑‑‑‑‑‑‑‑‑‑‑‑‑‑‑‑‑‑‑‑‑‑‑‑‑‑‑‑‑‑‑‑‑‑‑‑‑‑‑‑‑‑‑‑‑‑‑‑‑‑‑‑‑‑‑‑‑‑‑‑‑‑‑‑‑‑‑‑‑‑‑‑‑‑‑‑‑‑‑‑‑‑‑‑‑‑‑‑‑‑‑‑‑‑‑‑‑‑‑‑‑‑‑‑‑‑‑‑‑‑‑‑‑‑‑‑‑‑‑‑‑‑‑‑‑‑‑‑‑‑‑‑‑‑‑‑‑‑‑‑‑‑‑‑‑‑‑‑‑‑‑‑‑‑‑‑‑‑‑‑‑‑‑‑‑‑‑‑‑‑‑‑‑‑‑‑‑‑‑‑‑‑‑‑‑‑‑‑‑‑‑‑‑‑‑‑‑‑‑‑‑‑‑‑‑‑‑‑‑‑‑‑‑‑‑‑‑‑‑‑‑‑‑‑‑‑‑‑‑‑‑‑‑‑‑‑‑‑‑‑‑‑‑‑‑‑‑‑‑‑‑‑‑‑‑‑‑‑‑‑‑‑‑‑‑‑‑‑‑‑‑‑‑‑aggaagtcgaggaAGTCGCACGACAGAAAGAAATTGACCGCGCTAAGGCCCGTAAAGAACGTCACGAGGGGCGCTTAGAGGCACGCAGATTCAAACGTCGCAACCGCAAGGCACGTAAAGCACACAAAGCTAAGCGCGAAAGAATGCTTGCTGCGTGGCGATGGGCTGAACGTCAAGAACGGCGTAACCATGAGGTAGCTGTAGATGTACTAGGAAGAACCAATAACGCTATGCTCTGGGTCAACATGTTCTCTGGGGACTTTAAGGCGCTTGAGGAACGAATCGCGCTGCACTGGCGTAATGCTG  >  NC_001604.1/2737‑3029`  

                                                                                                                                                                                                                                                                                                                                                                                                                                                                                                                                                                                                                      
`CGTTTTCGACCACGCTTACGAAATGCTGAAAGAAAACATCCGTTATGATGACATCCGTGACACTGATGACCTGCACGATGCTATTCACATGGCTGCCGATAATGCAGTTCCGCACTACTACGCTGACATCTTTAGCGTAATGGCAAGTGAGGGCATTGACCTTGAGTTCGAAGACTCTGGTCTGATGCCTGACACCACGGACGTAATCCGCATCCTGCAAGCGCGTATCTATTAGCAATTAACGATTGACCTCTGGGAAGACGCAGAAGACTTGCCCAATGAATACTTGGAGGAAGTCGAG                                                                                                                                                                                                                                                                                                         >  1:73049/1‑301`  
 `GTTTTCGACCACGCTTACGAAATGCTGAAAGAAAACATCCGTTATGATGACATCCGTGACACTGATGACCTGCACGATGCTATTCACATGGCTGCCGATAATGCAGTTCCGCACTACTACGCTGACATCTTTAGCGTAATGGCAAGTGAGGGCATTGACCTTGAGTTCGAAGACTCTGGTCTGATGCCTGACACCAAGGACGTAATCCTCATCCTGCAAGCGCGTATCTATGAGCAATTAACGATTGACCTCTGGGAAGACGCAGAAGCCTTGCTCAATGAATACTTGGAGGAAGTCGAGG                                                                                                                                                                                                                                                                                                        >  1:457736/1‑301`  
 `GTTTTCGACCACGCGTACGAAATGCTGAAAGAAAACATCCGTTATGATGACATCCGTGACACTGATGACCTGCACGATGCTATTCACATGGCTGCCGATAATGCAGTTCCGCACTACTACGCTGACATCTTTAGCGTAATGGCAAGTGAGGGCATTGACCTTGAGTTCGAAGACTCTGGTCTGATGCCTGACACCAAGGACGTAATCCGCATCCTGCAAGCGCGTATCTATGAGCAATTAACGATTGACCTCTGGGAAGACGCAGAAGACTTGCTCAATGAATACTTGGAGGAAGTCGAGG                                                                                                                                                                                                                                                                                                        <  1:515776/301‑1`  
  `TTTTCGACCACGCTTACGAAATGCTGAAAGAAAACATCCGTTATGATGACATCCGTGACACTGATGACCTGCACGATGCTATTCACATGGCTGCCGATAATGCAGTTCCGCACTACTACGCTGACATCTTTAGCGTAATGGCAAGTGAGGGCATTGACCTTGAGTTCGAAGACTCTGGTCTGATGCCTGACACCAAGGACGTAATCCGCATCCTGCAAGCGCGTATCTATGAGCAATTAACGATTGACCTCTGGGAAGACGCAGAAGACTTGCTCAATGAATACTTGGAGGAAGTCGAGGA                                                                                                                                                                                                                                                                                                       <  1:390064/301‑1`  
   `ggagatgtgtataagagacagGCTGAAAGAAAACATCCGTTATGATGACATCCGTGACACTGATGACCTGCACGATGCTATTCACATGGCTGCCGATAATGCAGTTCCGCACTACTACGCTGACATCTTTAGCGTAATGGCAAGTGAGGGCATTGACCTTGAGTTCGAAGACTCTGGTCTGATGCCTGACACCAAGGACGTAATCCGCATCCTGCAAGCGCGTATCTATGAGCAATTAACGATTGACCTCTGGGAAGACGCAGAAGACTTGCTCAATGAATACTTGGAGGAAGTCGAGGAA                                                                                                                                                                                                                                                                                                      <  1:66905/280‑1`  
   `TTTCGACCACGCTTACGAAATGCTGAAAGAAAACATCCGTTATGATGACATCCGTGACACTGATGACCTGCACGATGCTATTCACATGGCTGCCGATAATGCAGTTCCGCACTACTACGCTGACATCTTTAGCGTAATGGCAAGTGAGGGCATTGACCTTGAGTTCGAAGACTCTGGTCTGATGCCTGACACCAAGGACGTAATCCGCATCCTGCAAGCGCGTATCTATGAGCAATTAACGATTGACCTCTGGGAAGACGCAGAAGACTTGCTCAATGAATACTTGGAGGAAGTCGAGGAA                                                                                                                                                                                                                                                                                                      <  1:218645/301‑1`  
   `TTTCGACCACGCTTACGAAAGGCTGAAAGAAAACATCCGTTATGATGACATCCGTGACACTGATGACCTGCACGATGCTATTCACATGGCTGCCGATAATGCAGTTCCGCACTACTACGCTGACATCTTTAGCGTAATGGCAAGTGAGGGCATTGACCTTGAGGTCGAAGACTCTGGTCTGATGCCTGACACCAAGGACGTAATCCGCATCCTGCAAGCGCGTATCTATGAGCAATTAACGATTGACATCTGGGAAGACGCAGAAGACTTGCTCAATGAATACTTGGAGGAAGTCGAGGAA                                                                                                                                                                                                                                                                                                      <  1:295801/301‑1`  
   `TTTCGACCACGCTAACGAAATGCTGAAAGAAAACATCCGTTATGATGACATCCGTGACACTGATGACCTGCACGATGCTATTCACATGGCTGCCGATAATGCAGTTCCGCACTACTACGCTGACATCGTTAGCGTAATGGCAAGTGAGGGCATTGACCTTGAGTTCGAAGACTCTGGTCTGATGCCTGACACCAAGGACGTAATCCGCATCCTGCAAGCGCGTATCTATGAGCAATTAACGATTGACCTCTGGGAAGACGCAGAAGACTTGCTCAATGAATACTTGGAGGAAGTCGAGGAA                                                                                                                                                                                                                                                                                                      <  1:189026/301‑1`  
    `TTCGACCACGCTTACGAAATGCTGAAAGAAAACATCCGTTATGATGACATCCGTGACACTGATGACCTGCACGATGCTATTCACATGGCTGCCGATAATGCAGTTCCGCACTACTACGCTGACATCTTTAGCGTAATGGCAAGTGAGGGCATTGACCTTGAGTTCGAAGACTCTGGTCTGATGCCTGACACCAAGGACGTAATCCGCATCCTGCAAGCGCGTATCTATGAGCAATTAACGATTGACCTCTGGGAAGACGCAGAAGACTTGCTCAATGAATACTTGGAGGAAGTCGAGGAAG                                                                                                                                                                                                                                                                                                     <  1:616102/301‑1`  
    `TTCGACCACGCTTACGAAATGCTGAAAGAAAACATCCGTTATGATGACATCCGTGACACTGATGACCTGCACGATGCTATTCACATGGCTGCCGATAATGCAGTTCCGCACTACTACGCTGACATCTTTAGCGTAATGGCAAGTGAGGGCATTGACCTTGAGTTCGAAGACTCTGGTCTGATGCCTGACACCAAGGACGTAATCCGCATCCTGCAAGCGCGTATCTATGAGCAATTAACGATTGACCTCTGGGAAGACGCAGAAGACTTGCTCAATGAATACTTGGAGGAAGTCGAGGAAG                                                                                                                                                                                                                                                                                                     <  1:111138/301‑1`  
          `CACGCTTACGAAATGCTGAAAGAAAACATCCGTTATGATGACATCCGTGACACTGATGACCTGCACGATGCTATTCACATGGCTGCCGATAATGCAGTTCCGCACTACTACGCTGACATCTTTAGCGTAATGGCAAGTGAGGGCATTGACCTTGAGTTCGAAGACTCTGGTCTGATGCCTGACACCAAGGACGTAATCCGCATCCTGCAAGCGCGTATCTATGAGCAATTAACGATTGACCTCTGGGAAGACGCAGAAGACTTGCTCAATGAATACTTGGAGGAAGTCGAGGAAGTCGCAC                                                                                                                                                                                                                                                                                               <  1:507714/301‑1`  
            `CGCTTACGAAATGCTGAAAGAAAACATCCGTTATGATGACATCCGTGATACTGATGACCTGCACGATGCTATTCACATGGCTGCCGATAATGCAGTTCCGCACTACTACGCTGACATCTTTAGCGTAATGGCAAGTGAGGGCATTGACCTTGAGTTCGAAGACTCTGGTCTGATGCCTGACACCAAGGACGTAATCCGCATCCTGCAAGCGCGTATCTATGAGCAATTAACGATTGACCTCTGGGAAGACGCAGAAGACTTGCTCAATGAATACTTGGAGGAAGTCGAGGAAGTCGCACGA                                                                                                                                                                                                                                                                                             >  1:529314/1‑301`  
            `CGCTTACGAAATGCTGAAAGAAAACATCCGTTATGATGACATCCGTGACACTGATGACCTGCACGATGCTATTCACATGGCTGCCGATAATGCAGTTCCGCACTACTACGCTGACATCTTTAGCGTAATGGCAAGTGAGGGCATTGACCTTGAGTTCGAAGACTCTGGTCTGATGCCTGACACCAAGGACGTAATCCGCATCCTGCAAGCGCGTATCTATGAGCAATTAACGATTGACCTCTGGGAAGACGCAGAAGACTTGCTCAATGAATACTTGGAGGAAGTCGAGGAAGTCGCACGA                                                                                                                                                                                                                                                                                             >  1:619617/1‑301`  
             `GCTTACGAAATGCTGAAAGAAAACATCCGTTATGATGACATCCGTGACACTGATGACCTGCACGATGCTATTCACATGGCTGCCGATAATGCAGTTCCGCACTACTACGCTGACATCTTTAGCGTAATGGCAAGTGAGGGCATTGACCTTGAGTTCGAAGACTCTGGTCTGATGCCTGACGCCAAGGACGTAATCCGCATCCTACAAGCGCGTATCTATGAGCAATTAACGATTGACCTCTGGGAAGACGCAGAAGACTTGCTCAATGAATACTTGGAGGAAGTCGAGGAAGTCGCACGAC                                                                                                                                                                                                                                                                                            <  1:250562/301‑1`  
             `GCTTACGAAATGCTACAAGAAAACATCCGTTATGATGACATCCGTGACACTGATGACCTGCACGATGCTATTCACATGGCTGCCGATAATGCAGTTCCGCACTACTACGCTGACATCTTTAGCGTAATGGCAAGTGAGGGCATTGACCTTGAGTTAGAAGACTCTGGTCTGATGCCTGACACCAAGGACGTAATCCGCATCCTGCAAGCGCGTATCTATGAGCAATTAACGATTGACCTCTGGGAAGACGCAGAAGACTTGCTCAATGAATACTTGGAGGAAGTCGAGGAAGTCGCACGAC                                                                                                                                                                                                                                                                                            <  1:478045/301‑1`  
               `TTACGAAATGCTGAAAGAAAACATCCGTTATGATGACATCCGTGACACTGATGACCTGCACGATGCTATTCACATGGCTGCCGATAATGCAGTTCCGCACTACTACGCTGACATCTTTAGCGTAATGGCAAGTGAGGGCATTGACCTTGAGTTCGAAGACTCTGGTCTGATGCCTGACACCAAGGACGTAATCCGCATCCTGCAAGCGCGTATCTATGAGCAATTAACGATTGACCTCTGGGAAGACGCAGAAGACTTGCTCAATGAATACTTGGAGGAAGTCGAGGAAGTCGCACGACAG                                                                                                                                                                                                                                                                                          <  1:82066/301‑1`  
                    `AAATGCTGAAAGAAAACATCCGTTATGATGACATCCGTGACACTGATGACCTGCACGATGCTATTCACATGGCTGCCGATAATGCAGTTCCGCACTACTACGCTGACATCTTTAGCGTAATGGCAAGTGAGGGCATTGACCTTGAGTTCGAAGACTCTGGTCTGATGCCTGACACCAAGGACGTAATCCGCATCCTGCAAGCGCGTATCTATGAGCAATTAACGATTGACCTCTGGGAAGACGCAGAAGACTTGCTCAATGAATACTTGGAGGAAGTCGAGGAAGTCGCACGACAGAAAGA                                                                                                                                                                                                                                                                                     <  1:459536/301‑1`  
                       `aagagacAGAAAACAACCGTTAGGATAACATCCGTGACACTGATGACCTGCACGATGCTATTCACAGGGATGCCGATAATGCAGTTCCGCACTACTACGCTGACATCTTTAGCGTAATGGAAAGTGAGGGCATTGACCTTGAGGTCGAAGACTCTGGTCTGATGCCTGACACCAAGGACGTAATCCGCATCCTGCAAGCGCGTATCTATGAGCAATTAACGATTGACCTCTGGGAAGACGCAGAAGACTTGCTCAATGAATACTTGGAGGAAGTCGAGGAAGTCGCACGACAGAAAGAAAT                                                                                                                                                                                                                                                                                  <  1:223838/294‑1`  
                       `TGCTGAAAGAAAACATCCGTTATGATGACATCCGTGACACTGATGACCTGCACGATGCTATTCACATGGCTGCCGATAATGCAGTTCCGCACTACTACGCTGACATCTTTAGCGTAATGGCAAGTGATGGCATTGACCTTGAGTTCGAAGACTCTGGTCTGATGCCTTACACCAAGGACGTAATCCGCATCCTGCAAGCGCGTATCTATGAGCAATTAACGATTGACCTCTGGGAAGACGCAGAAGACTTGCTCAATGAATACTTGGAGGAAGTCGAGGAAGTCGCACGACAGAAAGAAAT                                                                                                                                                                                                                                                                                  <  1:173265/301‑1`  
                       `TGCTGAAAGAAAACATCCGTTATGATGACATCCGTGACACTGATGACCTGCACGATGCTATTCACATGGCTGCCGATAATGCAGTTCCGCACTACTACGCTGACATCTTTAGCGTAATGGCAAGTGAGGGCATTGACCTTGAGTTCGAAGACTCTGGTCTGATGCCTGACACCAAGGACGTAATCCGCATCCTGCAAGCGCGTATCTATGAGCAATTAACGATTGACCTCTGGGAAGACGCAGAAGACTTGCTCAATGAATACTTGGAGGAAGTTGAGGAAGTCGCACGACAGAAAGAAAT                                                                                                                                                                                                                                                                                  <  1:540013/301‑1`  
                       `TGCTGAAAGAAAACATCCGTTATGATGACATCCGTGACACTGATGACCTGCACGATGCTATTCACATGGCTGCCGATAATGCAGTTCCGCACTACTACGCTGACATCTTTAGCGTAATGGCAAGTGAGGGCATTGACCTTGAGTTCGAAGACTCTGGTCTGATGCCTGACACCAAGGACGTAATCCGCATCCTGCAAGCGCGTATCTATGAGCAATTAACGATTGACCTCTGGGAAGACGCAGAAGACTTGCTCAATGAATACTTGGAGGAAGTCGAGGAAGTCGCACGACAGAAAGAAAT                                                                                                                                                                                                                                                                                  <  1:354053/301‑1`  
                       `TGCTGAAAGAAAACATCCGTGATGATGACATCCGTGACACTGATGACCTGCACGATGCTATTCACATGGCTGCCGATAATGCAGTTCCGCACTACTACGCTGACATCTTTAGCGTAATGGCAAGTGAGGGCATTGACCTTGAGTTCGAAGACTCTGGTCTGATGCCTGACACCAAGGACGTAATCCGCATCCTGCAAGCGCGTATCTATGAGCAATTAACGATTGACCTCTGGGAAGACGCAGAAGACTTGCTCAATGAATACTTGGAGGAAGTCGAGGAAGTCGCACGACAGAAAGAAAT                                                                                                                                                                                                                                                                                  <  1:603716/301‑1`  
                         `ataagagccagtATCCGTTATAATGCCATCCGTGCAACTTATGACCTGCACGATGCTATTCACATGGCTGCCGATAATGCAGTTCCGCACTACTACGCTGACATCTTTAGCGTAATGGCAAGTGAGGGCATTGACCTTGAGTTCGAAGACTCTGGTCTGATGCCTGACACCAAGGACGTAATCCGCATCCTGCAAGCGCGTATCTATGAGCAATTAACGATTGACCTCTGGGAAGACGCAGAAGACTTGCTCAATGAATACTTGGAGGAAGTCGAGGAAGTCGCACGACAGAAAGAAATTG                                                                                                                                                                                                                                                                                <  1:668073/289‑1`  
                         `CTGAAAGAAAACCTCCGTTATGATGACATCCGTGACACTGATGACCTGCACGATGCTATTCACATGGCTGCCGATAATGCAGTTCCGCACTACTACGCTGACATCTTTAGCGTAATGGCAAGTGAGGGCATTGACCTTGAGTTCGAAGACTCTGGTCTGATGCCTGACACCAAGGACGTAATCCGCATCCTGCAAGCGCGTATCTATGAGCAATTAACGATTGACCTCTGGGAAGACGCAGAAGACTTGCTCAATGAATACTTGGAGGAAGTCGAGGAAGTCGCACGACAGAAAGAAATTG                                                                                                                                                                                                                                                                                <  1:495910/301‑1`  
                           `aAAAGAAAACATCCGTTATGATGACATCCGTGACACTGATGACCTGCACGATGCTATTCACATGGCTGCCGATAATGCAGTTCCGCACTACTACGCTGACATCTTTAGCGTAATGGCAAGTGAGGGCATTGACCTTGAGTTCGAAGACTCTGGTCTGATGCCTGACACCAAGGACGTAATCCGCATCCTGCAAGCGCGTATCTATGAGCAATTAACGATTGACCTCTGGGAAGACGCAGAAGACTTGCTCAATGAATACTTGGAGGAAGTCGAGGAAGTCGCACGACAGAAAGAAATTGAC                                                                                                                                                                                                                                                                              <  1:367518/300‑1`  
                           `aAAAGAAAACATCCGTTATGATGACATCCGTAACACTGATGACCTGCACGATGCTATTCACATGGCTGCCGATAATGCAGTTCCGCACTACTACGCTGACATCTTTAGCGTAATGGCAAGTGAGGGCATTGACCTTGAGTTCGAAGACTCTGGTCTGATGCCTGACACCAAGGACGTAATCCGCATCCTGCAAGCGCGTATCTATGAGCAATTAACGATTGACCTCTGGGAAGACGCAGAAGACTTGCTCAATGAATACTTGGAGGAAGTCGAGGAAGTCGCACGACAGAAAGAAATTGAC                                                                                                                                                                                                                                                                              <  1:543909/300‑1`  
                           `aAAAGAAAACATCCGTTATGATGACATCCGGGACACTGATGACCTGCACGAGGCTATTCACATGGCTGCCGATAATGCAGTTCCGCACTACTACGCTGACATCTTTAGCGTAATGGCAAGTGAGGGCATTGACCTTGAGTTCGAAGACTCTGGTCTGATGCCTGACACCAAGGACGTAATCCGCATCCTGCAAGCGCGTATCTATGAGCAATTAACGATTGACCTCTGGGAAGACGCAGAAGACTTGCTCAATGAATACTTGGAGGAAGTCGAGGAAGTCGCACGACAGAAAGAAATTGAC                                                                                                                                                                                                                                                                              <  1:654200/300‑1`  
                           `GAAAGAAAACATCCGTTATGATGACATCCGTTACACTGATGACCTGCACGATGCTATTCACATGGATGCCGATAATGCAGTTCCGCACTACTACGCTGACATCTTTAGCGTAATGGCAAGTGAGGGCATTGACCTTGAGTTCGAAGACTCTGGTCTGATGCCTGACACCAAGGACGTAATCCGCATCCTGCAAGCGCGTATCTATGAGCAATTAACGATTGACCTCTGGGAAGACGCAGAAGACTTGCTCAATGAATACTTGGAGGAAGTCGAGGAAGTCGCACGACAGAAAGAAATTGAC                                                                                                                                                                                                                                                                              <  1:342419/301‑1`  
                           `GAAAGAAAACATCCGTTATGATGACATCCGTGACACTGATGACCTGCACGATGCTATTCACATGGCTGCCGATAATGCAGTTCCGCACTACTACGCTGACATCTTTAGCGTAATGGCAAGTGAGGGCATTGACCTTGAGTTCGAAGACTCTGGTCTGATGCCTGACACCAAGGACGTAATCCGCATCCTGCAAGCGCGTATCTATGAGCAATTAACGATTGACCTCTGGGAAGACGCAGAAGACTTGCTCAATGAATACTTGGAGGAAGTCGAGGAAGTCGCACGACAGAAAGAAATTGAC                                                                                                                                                                                                                                                                              <  1:138058/301‑1`  
                           `GAAAGAAAACATCCGTTATGATGACATCCGGGACACTGATGACCGGCACGATGCTAGTCAGATGGCGGCCGATAATGCAGTGCCGCACTACGACGCTGACATCTTTAGCGTAATGGCAAGTGAGGGCATGGACCTTGAGTTCGAAGACTCTGGTCTGATGTCTGACACCAAGGACGTAATCCGCATCCTGCAAGCGCGTATCTATGAGCAATTAACGATTGACCTCTGGGAAGACGCAGAAGACTTGCTCAATGAATACTTGGAGGAAGTCGAGGAAGTCGCACGACAGAAAGAAATTGAC                                                                                                                                                                                                                                                                              <  1:427819/301‑1`  
                               `aAAAACATCCGTTATGATGACATCCATGAAACTGATGACCTGCACGATGCTATTCACATGGCTGCCGATAATGCAGTTCCGCACTACTACGCTGACATCTTTAGCGTAATGGCAAGTGAGGGCATTGACCTTGAGTTCGAAGACTATGGTCTGATGCCTGACACCAAGGACGTAATCCGCATCCTGCAAGCGCGTATCTATGAGCAATTAACGATTGACCTCTGGGAAGACGCAGAAGACTTGCTCAATGAATACTTGGAGGAAGTCGAGGAAGTCGCACGACAGAAAGAAATTGACCGCG                                                                                                                                                                                                                                                                          <  1:385773/300‑1`  
                               `GAAAACATCCGTTATGATGACATCCGTGACACTGATGACCTGCACGATGCTATTCACATGGCTGCCGATAATGCAGTTCCGCACTACTACGCTGACATCTTTAGCGTAATGGCAAGTGAGGGCATTGACCTTGAGTTCGAAGACTCTGGTCTGATGCCTGACACCAAGGACGTAATCCGCATCCTGCAAGCGCGTATCTATGAGCAATTAACGATTGACCTCTGGGAAGACGCAGAAGACTTGCTCAATGAATACTTGGAGGAAGTCGAGGAAGTCGCACGACAGAAAGAAATTGACCGCG                                                                                                                                                                                                                                                                          >  1:202605/1‑301`  
                                     `acCCGTTATGATGACAGCCGTCACACTGATTACCTGCACGATGCTATTAACATGGTAGCCGATAATGCAGTTCCGCACTACTACGCTGACATCGTTAGCGTAATGGCAAGTGAGGGCATGGACCTTGAGTTCGAAGACTCTGGTCTGATGCCTGACACCAAGGACGTAATCCGTATCCTGCAAGCGCGTATCTATGAGCAATTAACGATTGACCTCTGGGAAGACGCAGAAGACTTGCTCAATGAATACTTGGAGGAAGTCGAGGAAGTCGAACGACAGAAAGAAATTGACCGCGCTAAGG                                                                                                                                                                                                                                                                    <  1:227888/299‑1`  
                                            `ATGATGGCATCCGTGACACTGATGACCTGCACGATGCTATTCACATGGCTGCCGACAATGCAGTTCCGCACTACTACGCTGACATCTTTAGCGTAATGGCAAGTGAGGGCATTGACCTTGAGTTCGAAGACTCTGGTCTGATGCCTGACACCAAGGACGTAATCCGCATCCTGCAAGCGCGTATCTATGAGCAATTAACGATTGACCTCTGGGAAGACGCAGAAGACTTGCTCAATGAATACTTGGAGGAAGTCGAGGAAGTCGCACGACAGAAAGAAATTGctgtctcttatacacatct                                                                                                                                                                                                                                                             >  1:640917/1‑282`  
                                                                       `tggACGATGCTATTCGAATGGCGGCCGATAAGGCAGTTCCGAACTACTGCGCCAACAACTTGAGCTTAACGGCAAGTGAGGGCATTGAACTTGAGTTCGAATACTCTGGTCGGATGCCTGAGACCAAGGACGTAAGCCGCATCCTGCAAGGGCGAATCTATGAGCAATTAACGATTAACCTCTGGGAAGACGCAGAAGACTTGCTCAATGAATACTTGGAGGAAGTCGAGGAAGTCGCACGACAGAAAGAAATTGACCGCGCTAAGGCCCGTAAAGAACGTCACGAGGGG‑GCTTAGAGGCA                                                                                                                                                                                                                                 <  1:65517/298‑1`  
                                                                                     `CACATGGCTGACGATAATGCAGTTCCGCACTACTACGCAGACAGCGGGAGCGGAATGGCAAGTGAGGGCATGGAGAGTGAGTTCGAAGACTCTGGGCTGATGCCTGACACCAAGGACATAAACCGGATCCTGCAAGCAGATATCAATGGGCAGTTAACGATTGAACTCTGGGAAGAAGAAGAAGACTTGCTCAAGGAATACTTGGAGGAAGTCGAGGAAGTCGGACGACAGAAAGAAATTGACCGCGCTAAGGCCCGTAAAGAACGTCACGAGGGGCGCTTAGAGGCACGCAGATTCAAAC                                                                                                                                                                                                                    <  1:36219/301‑1`  
                                                                                                                    `ACTACGCTGACATCTTTAGCGTAATGGCAAGTGAGGGCATTGTCTTTGAGTTCGAAGACTCTGTTCTGATGCCTGCCACCAAGGACGTAATCCGCATCCTGCGAGCGCGTATCTCTGAGCAATTAACGATTGACCTCTGGGAAGACGCAGAAGACTTGCTCGATGAATACTTGGAGTAAGTCGAGGAAGTCGCCCGCCAGAAGGAAATTGACCGCGATAAGGCCCGTAAAGGACGTCACGAGTGGCGTTTCGAGGCCCGCCGATTAAACCTGTGCAACAGACAAGCACGTGACGCATACAA                                                                                                                                                                                     >  1:314834/1‑301`  
                                                                                                                                   `gagatgtgtataagagacAGGGAATTGACCTTGAGTTCGAAGCCGCTGGTTTGATGCCTGAAACCAAGGACGAAAGCCGCATCCTGCAAGCGCGTATCTATGAGCAATTAACGAGTGACTTCTGGGAAGACACAGAAGACTTGCTAAATGAATACTTGGAGGAAGGCGAGGAAGTCGCACGACAGAAAGAAATTGACCGCGCTAAGGCCCGTAAAGAACGTCACGAGGGGCGCTTAGAGGAACGCAGATTCAAACGTCGCAACCGCAAGGCACGTAAAGCACACAAAGCTAAGCGCGAAAG                                                                                                                                                                      <  1:627399/283‑1`  
                                                                                                                                         `aAATGGAAAGGGAGGACATTGACCATGAGTTCGAAGACTTTGGTCTGATGCCTGACACAAAGGACGTAAAACGACTGCTGCAAGCGCGTATCTATGAGCAATTAACGAAGGACCTCTGGGAAGACGCAGAAGACTTGCTCAATGATAACTTGGAGGAAGTCGAGGAAGTCGAACGACAGAAAGAAATTGACCGCGCTAAGGCCCGTAAAGAACGTCACGAGGGGCGCTTAGAGGCACGCAGATTCAAACGTCGCAACCGTAAGGCACGGAAAGCACACAAAGCTAAGCGCGAAAGAATGCT                                                                                                                                                                <  1:14628/300‑1`  
                                                                                                                                                                         `GAAGACTCTGGTGTGATTGATAACAACCAGGACGTAAGTCTCCTCCTACAAGCGCGTATCTATTATCAATTAACGATTGACCTCTGAGAAGGCGCAGAAGACTGGCTCAATGAATACTTGGGGGAAGTCGAGGACGTCGCACGACGGAAAGAAATTGACCGCGCTCAGGCCCGTAAAGAACGTCACGAGGGGCGCTTAGAGGCACGCAGATTCAACCGTCGCAACCGCAAGGCACGTAAAGCACACAAAGCTAAGCGCGAAAGAATGCTTGCTGCGTGGCGATGGGCTGAACGTCAAGAAC                                                                                                                                <  1:588843/301‑1`  
                                                                                                                                                                              `aaaaggtcgtaaGCCAGACAACAAGGACATAACCTGCATCCTGCAAGCTCGTATCGATGAGCAATGAACGATTGACCTCGGGGAAGACGCAGAAGACGTGCTCAATGAATACGTGGAGAAAGTCGAGGAAGTCGCACAACATAAAGAAAGTGACCGCGCTAAGGCCCGTAAAGAACGTCACGAGGGGCGCTTAGAGGCACGCAGATTCAAACGTCGCAACCGCAAGGCACGTAAAGCACACAAAGCTAAGCGCGAAAGAATGCTTGCTGCGTGGCGATGGGCTGAACGTCAAGAACGGCGT                                                                                                                           <  1:162021/289‑1`  
                                                                                                                                                                                 `ggtggatcggagatgggtataagagacagTCCGCATCCTGCAAGCGCGTAGCTATGAGCAATTAACGATGGACCTGTGGGAAGACGCAGAAGACTTGCTCAATGAATACGTGGAGGAAGTGGAGGAAGTCGCACGAGAGAAAGAAATTGACCGAGCTAAGGACCGTAAAGAACGTCACGAGGGACGCTTAGAGGAACGCAGATTCAAACGTCGCAACCGCAAGGCACGTAAAGCACACAAAGCTAAGCGCGAAAGAATGCTTGCTGCGTGGCGATGGGCTGAACGTCAAGAACGGCGTAAC                                                                                                                        <  1:74202/272‑1`  
                                                                                                                                                                                        `ATGACTGAAACAAAGGATGTAGACCGGAGCCTGCAAGGGCGTATCTATGAGCAAGTAACGAATGACCTCTGGGAAGACGCAGAAAACGTGCTAAAGGAATACTTGGAGGAAGCGGAGGAAGGCGCACAAAAAAAAGAAACTGACCGCGATAAGGCCCGTAAAGAACGTCACGAGGGGCGCTTAGAGGTACGCAGATTAAAACGTCGCAACCGCAAGGGACGGAAAGCACACAAAGCTAAGCGCGAAAGAATGCTTGCTGCGTGGAGATGGGCTGAACGTCAAGAACGGCGTAACCATGAGG                                                                                                                 <  1:395651/301‑1`  
                                                                                                                                                                                                  `CCAAGGACGAAATCATCATGCTGCAAGCGCGTATCTATGAGCAATTAACTAATGACCGCTGGGAAGGCGCAGAAGTCTTGCTCAAAGAATACTTGGAGGAAGTCGAGGAAGTCGGACGACATAAACAAATTGACCGAGCTAAGGCCCGTAAAGAACGTCACGAGGGGCGCTTAGAGGGACGCAGATTCAAACGTCGCAACCGCAAGGCACGTAAAGCACACAAAGCTAAGCGCGAAAGAATGCTTGCTGCGTGGAGATGGGCTGAAC‑‑‑‑‑GAACGGCGTAACCATGAGGTATCTGTAGATGTAC                                                                                                  <  1:199760/301‑1`  
                                                                                                                                                                                                            `AATCCTCATCCTGCAACCGCCGATCAATGATCAAGTAACGATTGACCCCTGGGAAGACGCAGAAGACTTGCTAAATGACTACTTGGAGGAAGTCGACGAAGTCGCTCGACATAAAGAAATTTACCGCCCCAAGGCCCGTAAAGAACTTCACTAGGGGCGCTTAGAGGCACGCAGATTCAAACGTCTCAACCGCAATGCACGTAAAGCACACAAAGCTAAGCGCGAAAGAATTCTTGCTGCGTGGCGATGGTCTGAACGTCAAGAACGGCGTAACCATGAGGTAGCTGTAGATGTACTAGGA                                                                                             <  1:178752/301‑1`  
                                                                                                                                                                                                            `AATCCAGATCCTGATAGCGGGTATTTAAGGACAATTAACGAATGACCTCTGGGAAGACGCAGAAGTGTTGCAGAATGAATACTTGGAGGAAGTCGAGGAAGGCGCACGACAGAAAGAAATTGACCGCGCTAAGGACCGTAAAGAACGTCACGAGGGACGCTTAGAGGCACGAAGAGTCAAACGTCGCAAACGCAAGGCACGTAAAGCACACAACGCTAAGCGGGAAAGAATGCTTGCTGCGTGGAGAAGGGCTGAACGTCAAGAACGGCGTAACCATGAGGTAGCTGTAGATGTACTAGGA                                                                                             <  1:58674/301‑1`  
                                                                                                                                                                                                                           `agtcttgttcgatttGCAATTAAGGATTGACCTTTGGTAAGCCGGAGATTGCTTGGTAAAGGAATCATTGGTTGAAGTCAAGGAAGTCGCACGACAGAAAGATATTGACCGCGCTAAGGCCCGTAAAGACCGTCACGAGGGGCGCTTAGAGGCACGCAGATTCAAACGTCGCAACCGCAGGGCGCTTAAAGCACACAAAGCTAAGCGGGAAAGAATGCTTGCTGCGTGGCGATGGGCTGAACTTCAAGATCGGCGTAACCATGAGGTAGCTGTAGATGTACTAGGAAGAACCAATAACGCT                                                                              <  1:558809/286‑1`  
                                                                                                                                                                                                                                                     `tgGACCTCTGGGAAGACGCAGAAGAGCTGCAAAATGAATACCTGGAGGAAGTCGAGGAAGTCGCACGACAGAAAGAAATTGACCGCGCTAAGGCCCGTAAAGAACGTCACGAGGGGCGCTTAGAGGCACGCAGATTCAAACGTCGCAACCGCAAGGCACGTAAAGCACACAAAGCTAAGCGCGAAAGAATGCTTGCTGCGTGGCGATGGGCTGAACGTCAAGAACGGCGTAACCATGAGGTAGCTGTAGATGTACTAGGAAGAACCAATAACGCTATGCTCTGGGTCAACATGTTCTCTGG                                                    <  1:436376/299‑1`  
                                                                                                                                                                                                                                                     `aaagccgcgGGGAAAAAGCAGAAAACTTGCTCGATGAAGAATTGGAGGAAGTGGAGGAAGTCGCACGACAGAAAGAAATTGACAGCGCTAAGGACCGTAAAGAACGTCACGAGGGGCGCTTAGAGGCACGCAGATTCAAACGTCGCAACCGCAAGGCACGTAAAGCACACAAAGCTAAGCGCGAAAGAATGCTTGCTGCGTGGCGATGGGCTGAACGTCAAGAACGGCGTAACCATGAGGTAGCTGTAGATGTACTAGGAAGAACCAATAACGCTATGCTCTGGGTCAACATGTTCTCTGG                                                    <  1:426224/292‑1`  
                                                                                                                                                                                                                                                       `gggggctcggagttgtgaataagagacagCAAAGAATACTTGGAGGAAGTCGAGGAAGTCGCACGACAGAAAGAAATTGACCGCTCGAAGGCCCGTAAAGAACGTCACGAGGGGCGCTTAGAGGCACGCAGATTCAAACGTCGCAACCGCAAGGCACGTAAAGCACACAAAGCTAAGCGCGAAAGAATGCTTGCTGCGTGGCGATGGGCTGAACGTAAAGAACGGCGTAACCATGAGGTAGCGGTAGATGTACTAGGAAGAACCAATAACGCTATGCTCTGGGTCAACATGTGCTCTGGGG                                                  <  1:437970/272‑1`  
                                                                                                                                                                                                                                                         `CCTCTTGGAAGCCTCAGAAGACGTCCTCAATGAATACTTGGAGGAAGTCGAGGACGTCGCACGACAGAAAGAAATTTACCACGCTAAGGCCCGTAAAGAACGTCACGGGTGGCGCTTAGAGCCACTCAGATTCAAACGTCGCAACCGCAAGGCACGTAAATCACACAAAGCTAAGCGCGAAAGAATGCTTGCTGCGTGGCGATGGGCTGAACGTCAAGAACGGCGTAACCATGAGGTATCTGTAGATGTACTAGGAAGAACCAATAACGCTATGCTCTGGGTCAACATGTTCTCTGGGGAC                                                <  1:124543/301‑1`  
                                                                                                                                                                                                                                                            `ctcggagatgtgtataagagacagCAATGAATACTTGGAGGAAGTCGAGGAAGTCGCACGACAGAAAGAAATTGACCGCGCTAAGGCCCGTAAAGAACGTCACGAGGGGCGCTTAGAGGCACGCAGATTCAAACGTCGCAACCGCAAGGCACGTAAAGCACACAAAGCTAAGCGCGAAAGAATGCTTGCTGCGTGGCGATGGGCTGAACGTCAAGAACGGCGTAACCATGAGGTAGCTGTAGATGTACTAGGAAGAACCAATAACGCTATGCTCTGGGTCAACATGTTCTCTGGGGACTTT                                             <  1:560857/277‑1`  
                                                                                                                                                                                                                                                                 `ggaagagtgtaaaagggacagATGAATACTTGGGAGAAGTCGAGGAAGTCGCACGACAGAAAGAAATTGACCGCGCTAAGGCCCGTAAAGAACGTCACGAGGGGCGCTTAGAGGCACGCAGATTCAAACGTCGCAACCGCAAGGCACGTAAAGCACACAAAGCTAAGCGCGAAAGAATGCTTGCTGCGTGGCGATGGGCTGAACGTCAAGAACGGCGTAACCATGAGGTAGCTGTAGATGTACTAGGAAGAACCAATAACGCTATGCTCTGGGTCAACATGTTCTCTGGGGACTTTAAGGC                                        <  1:514266/280‑1`  
                                                                                                                                                                                                                                                                   `GACGCAGAAGACTTGCGCAATGAATATTTGGAGGAAGTCGAGGAAGTCGCACGACAGAAAGAAATTGACCGCTCTAAGGCCCGTAAAGAACGTCACGAGGGGCGCTTAGAGGCACGCAGATTCAAACGTCGCACCCGCAAGGCACGTAAAGCACACAAAGCTAAGAGCGAAAGAAAGCTTGCTGCGTGGAGAAGGGCTGAAAGTAAAGAACGGAGTAATCATGAGGTAGCTGAAGCTGTAATATGAAGAACCAATAAAGATATGCTCTGGGTAAACATATTATCTGGGGACTTTAAGGAGC                                      >  1:238605/1‑301`  
                                                                                                                                                                                                                                                                    `ACGCAGAAGACTTGCTCAATGAATACTTGGAGGAAGTCGAGGAAGTCGCACGACAGAAAGAAATTGACCGCGCTAAGGCCCGTAAAGAACGTCACGAGGGGCGCTTAGAGGCACGCAGATTCAAACGTCGCAACCGCAAGGCACGTAAAGCACACAAAGCTAAGCGCGAAAGAATGCTTGCTGCGTGGCGATGGGCTGAACGTCAAGAACGGCGTAACCATGAGGTAGCTGTAGATGTACTAGGAAGAACCAATAACGCTATGCTCTGGGTCAACATGTTCTCTGGGGACTTTAAGGCGCT                                     >  1:20312/1‑301`  
                                                                                                                                                                                                                                                                       `CAGAAGACTTGCTCAATGAATACTTGGAGGAAGTCGAGGAAGTCGCACGACAGAAAGAAATTGACCGCGCTAAGGCCCGTAAAGAACGTCACGCGGGGCGCTTAGAGGCACGCAGATTCAAACGTCGCAACCGCAAGGCACGTCAAGCACACAAAGCTAAGCGCGAAAGAATGCGTGCTGCGTGGCGATGGGCTGAACGTCAAGAACGGAGTAACCATGAGGTAGCTGAAGATGTACTAGGAAGAACCAATAACGCTAAGCTCTGGGTCAACATGTTATCTGAGGACCTTctgtatcttat                                  >  1:599571/1‑290`  
                                                                                                                                                                                                                                                                           `aagagacagCAATGAATACTTGGAGGAAGTCGAGGAAGTCGCACGACAGAAAGAAATTGACCGCGCTAAGGCCCGTAAAGAACGTCACGAGGGGCGCTTAGAGGCACGCAGATTCAAACGTCGCAACCGCAAGGCACGTAAAGCACACAAAGCTAAGCGCGAAAGAATGCTTGCTGCGTTGCGATGGGCTGAACGTCAAGAACGGCGTAACCATGAGGTAGCTGTAGATGTACTATGAAGAACCAATAACGCTATGCTCTGGGTCAACATGTTCTCTGGGGACTTTAAGGCGCTTGAGGAA                              <  1:617429/292‑1`  
                                                                                                                                                                                                                                                                           `AGAGTTGCTCAATGAATACTTGGAGGAAGTCGAGGAAGTCGCACGACAGAAAGAAATTGACCGCGCTAAGGCCCGTAAAGAACGTCACGAGGGGCGCTTAGAGGCACGCAGATTCAAACTTCGCAACCGCCAGTCACGTAAAGCCCACAAAGCTAAGCTCGAAAGAATGCTTGCTGCGTGGCGATGGGCTGAACGTCCAGAACGGCGTAACCATGAGGTAACTTTAGATGTACTAGGAAGAACCAATAACGCTATGCTCTGGGTCAACATGTTCTCTGGGTACTTTAAGGCGCTTCAGTAA                              >  1:110656/1‑301`  
                                                                                                                                                                                                                                                                            `GACTTGCTCAATGAATACTTGGAGGAAGTCGAGGAAGTCGCACGACAGAAAGAAATTGACCGCGCTAAGGCCCGTAAAGAACGTCACGAGGGGCGCTTAGAGGCACGCAGATTCAAACGTCGCAACCGCAAGGCACGTAAAGCACACAAAGCTAAGCGCGAAAGAATGCTTGCTGCGTGGCGATGGGCTGAACGTCAAGAACGGCGTAACCATGAGGTAGCTGTAGATGTACTAGGAAGAACCAATAACGCTATGCTCTGGGTCAACATTTTCTCTGGGGACTTTAAGTCGCTTGAGGAAC                             >  1:85137/1‑301`  
                                                                                                                                                                                                                                                                              `tgtataagagacagACTGGGAGGAAGTCGAGGAAGTCGCACGACAGAAAGAAATTGACCGCGCTAAGGCCCGTAAAGAACGTCACGAGGGGCGCTTAGAGGCACGCAGATTCAAACGTCGCAACCGCAAGGCACGTAAAGCACACAAAGCTAAGCGCGAAAGAATGCTTGCTGCGTGGCGATGGGCTGAACGTCAAGAACGGCGTAACCATGAGGTAGCTGTAGATGTACTAGGAAGAACCAATAACGCTATGCTCTGGGTCAACATGTTCTCTGGGGACTTTAAGGCGCTTGAAGAACGA                           <  1:581270/287‑1`  
                                                                                                                                                                                                                                                                              `aTTGCTCAATTAATACTTGGAGGAAGTCTAGGAATTCGACCGACAGAAAGAAATTGACCGCGCTAAGGCCCGTAAAGAACGTCACGAGGGGCGCTTAGAGGCACGCAGATTCAAACGTCGCAACCGCAAGGCACGTAAAGCACACAAAGCTAAGCGCGAAAGAATGCTTGCTGCGTGGCGATGGGCTGAACGTCAAGAACGGCGTAACCATGAGGTAGCTGTAGATGTACTAGGAAGAACCAATAACGCTATGCTCTGGGTCAACATGTTCTCTGGGGACTTTAAGGCGCTTGAGGAACGA                           <  1:331254/300‑1`  
                                                                                                                                                                                                                                                                                 `GCTCAATGAATACTTGGAGGAAGTCGAGGAAGTCGCACGACAGAAAGAAATTGACCGCGCTAAGGCCCGTAAAGAACGTCACGAGGGGCGCTTATAGGCACGCAGATTCAAACGTCGCAACCGCAAGGCACGTAAAGCACACAAAGCTAAGCGCGAAAGAATGCTTGCTGCGTGGCGATGGGCTGAACGTCAAGAACGGCGTAACCATGAGGTAGCTGTAGATGTACTAGGAAGAACCAATAACGCTATGCTCTGGGTCAACATGTTCTCTGGGGACTTTCAGGCGCTTTAGGAACGAATC                        >  1:85720/1‑301`  
                                                                                                                                                                                                                                                                                 `GCTCAATGAATACTTGGAGGAAGTCGAGGAAGTCGCACGACAGAAAGAAATTGACCGCGCTAAGGCCCGTAAAGAACGTCACGAGGGGCGCTTAGAGGCACGCAGATTCAAACGTCGCAACCGCAAGGCACGTAAAGCACACAAAGCTAAGCGCGAAAGAATGCCTGCTGCGTGGCGATGGGCTGAACGTCCAGAACGGCGTAACCATGAGGTAGCTGTAGATGTACTAGGAAGCACCAATAAAGCTATGCTCTGGGTCAACATGTTCTCTGGGGACTTTAAGGAGCTTGAGGACCGAATC                        >  1:338284/1‑301`  
                                                                                                                                                                                                                                                                                  `CTCAATGAATACTTGGAAGAAGTCGAGGAAGTCGCAAGACAGAAAAGAATTGACCGCGCTAAGGCCCGTAAAGAACGTCACGAGGGGCGCTTAGAGGCACGCAGATTCAAACGTCGCAACCGCAAGGCACGTAAAGCACACAAAGCTAAGCGAGAAAGAATGCTTGCTGCGTGGCGATGGGCTGAACGTCAAGAACGGCGTAACCATGAGGTAGCTGTAGATGTACTAGGAAGAACCAATAACGCTATGCTCTGGGTCAACATGTTCTCTGGGGACTTTAAGGCGCTTGAGGAACGAATCG                       <  1:638719/301‑1`  
                                                                                                                                                                                                                                                                                    `CAATGAATACTTGGAGGAAGTCGAGGAAGTCGCACGACAGAAAGAACTTGACCGAGCTAAGGCCCGTAAAGAACGTCACGAGGGGCGCTTAGAGGCACGCAGATTCAAACGTCGCAACCGCAAGGCACGTAAAGCACACAAAGCTAAGCGCGAAAGAATGCTTGCTGCGTGGCGATGGGCTGAACGTCAAGAACGGCGTAACCATGAGGTAGCTGTAGATGTACTAGGAAGAACCAATAACGCTATGCTCTGGGTCAACATGTTCTCGTGGGACTTTCAGGTGCTTTAGGAACGAAACGCG                     >  1:100281/1‑301`  
                                                                                                                                                                                                                                                                                    `CAATGAATACTTGGAGGAAGTCGAGGAAGTCGCACGACAGAAAGAAATTGACCGCGCTAAGGCCCGTAAAGAACGTCACGAGGGGCGCTTAGAGGCACGCAGATTCAAACGTCGCAACCGCAAGGCACGTAAAGCACACAAAGCTAAGCGCGAAAGAATGCTTGCTGCGTGGCGATGGGCTGAACGTCAAGAACGGCGTAACCATGAGGTAGCTGTAGATGTACTAGGAAGAACCAATAACGCTATGCTCTGGGTCAACATGTTCTCTGGGGACTTTAAGGCGCTTGAGGAACtgtctctt                     >  1:217014/1‑293`  
                                                                                                                                                                                                                                                                                    `CAATGAATACTTGGAGGAAGTCGAGGAAGTCGCACGACAGAAAGAAATTGACCGCGCTAAGGCCCGTAAAGAACGTCACGAGGGGCGCTTAGAGGCACGCAGATTCAAACGTCGCAACCGCAAGGCACGTAAAGCACACAAAGCTAAGCGCGAAAGAATGCTTGCTGCGTGGCGATGGGCTGAACGTCAAGAACGGCGTAACCATGAGGTAGCTGTAGATGTACTAGGAAGAACCAATAACGCTATGCTCTGGGTCAACATGTTCTCTGGGGACTTTAAGGCGCTTGAGGAACGAATCGCc                     >  1:517651/1‑300`  
                                                                                                                                                                                                                                                                                    `CAATGAATACTTGGAGGAAGTCGAGGAAGTCGCACGACAGAAAGAAATTGACCGCGCTAAGGCCCGTAAAGAACGTCACGAGGGGCGCTTAGAGGCACGCAGATTCAAACGTCGCAACCGCAAGGCACGTAAAGCACACAAAGCTAAGCGCGAAAGAATGCTTGCTGCGTGGCGATGGGCTGAACGTCAAGAACGGCGTAACCATGAGGTAGCTGTAGATGTACTAGGAAGAACCAATAACGCTATGCTCTGGGTCAACATGTTCTCTGGGGACTTTAAGGCGCTTGAGGAACGAATCGCG                     >  1:525008/1‑301`  
                                                                                                                                                                                                                                                                                    `CAATGAATACTTGGAGGAAGTCGAGGAAGTCGCACGACAGAAAGAAATTGACCGCGCTAAGGCCCGTAAAGAACGTCACGAGGGGCGCTTAGAGGCACGCAGATTCAAACGTCGCAACCGCAAGGCACGTAAAGCACACAAAGCTAAGCGCGAAAGAATGCTTGCTGCGTGGCGATGGGCTGAACGTCAAGAAAGGCGTAACCATGAGGTAGCTGTAGATGTACTAGGAAGAACCAATAACGCTATGCTCTGGGTCAACATGGTCTCAGGGGAATTTAAGGCGATTGAGGCACtgtctctt                     >  1:647565/1‑293`  
                                                                                                                                                                                                                                                                                     `AATGAATACTTGGAGGAAGTCGAGGAAGTCGCACGACAGAAAGAAATTGACCGCGCTAAGGCCCGTAAAGAACGTCACTAGGGGCGCTTAGAGGCACGCAGATTCAAACGTCGCAACCGCAAGGCACGTAAAGCACACAAAGCTAAGCGCGAAAGAATGCTTGCTGCGTGGCGATGGGCTGAACGTCAAGAACGGCGTAACCATGAGGTAGCTGTAGATGTACTAGGAAGAACCAATAACGCTATGCTCTGGGTCAACATGTTCTCTGGGGACTTTAAGGCGCTTGAGGAACGAATCGCGC                    >  1:118450/1‑301`  
                                                                                                                                                                                                                                                                                      `ATGAATACTTGGAGGAAGTCGAGGAAGTCGCACGACAGAAAGAAATTGACCGCGCTAAGGCCCGTAAAGAACGTCACGAGGGGCGCTTAGAGGCACGCAGATTCAAACGTCGCAACCGCAAGGCACGTAAAGCACACAAAGCTAAGCGCGAAAGAATGCTTGCTGCGTGGCGATGGGCTGAACGTCAAGAACGGCGTAACCATGAGGTAGCTGTAGATTTACTAGGAAGAACACATAACGCTATGCTCTGGGTCAACATGTTCTCTGGGGCCTTTAAGGAGCTTGAGGAACGAATCGCGag                   >  1:327408/1‑299`  
                                                                                                                                                                                                                                                                                      `ATGAATACTTGGAGGAAGTCGAGGAAGTCGCACGACAGAAAGAAATTGACCGCGCTAAGGCCCGTAAAGAACGTCACGAGGGGCGCTTAGAGGCACGCAGATTCAAACGTCGCAACCGCAAGGCACGTAAAGCACACAAAGCTAAGCGCGAAAGAATGCTTGCTGCGTGGCGATGGGCTGAACGTCAAGAACGGCGTAACCATGAGGTAGCTGTAGATGTACTAGGAAGAACCAATAACGCTATGCTATGGGTCAACATGTTTTCTGGGGACTTTAAGGCGCTTAAGGGACGCATAGCGCc                   >  1:205890/1‑300`  
                                                                                                                                                                                                                                                                                      `ATGAATACTTGGAGGAAGTCGAGGAAGTCGCACGACAGAAAGAAATTGACCGCGCTAAGGCCCGTAAAGAACGTCACGAGGGGCGCTTAGAGGAACGCAGATTCAAACGTCGCAACCGCAAGGCACGTAAAGCACACAAAGCTAAGCGCGAAAGAATGCTTGCTGCGTGGCGATGGGCTGAACGTCAAGAACGGCGTAACCATGAGGTAGCTGTAGATGTACTAGGAAGAACCAATAACGCTATGCTCTGGGTCAACATGTTCTCTGGGGACTTTAAGGCGCTTGAGGAACGAATCGCGCT                   >  1:153126/1‑301`  
                                                                                                                                                                                                                                                                                      `ATGAATACTTGGAGGAAGTCGAGGAAGTCGCACGACAGAAAGAAATTGACCGCGCTAAGGCCCGTAAAGAAAGTCACGAGGGGCGCTTAGAGGCACGCAGATTCAAACGTCGCAACCGCAAGGCACGTAAAGCACACAAAGCTAAGCGCGAAAGAATGATTGCTGCGTGGCGATGGGCTGAACGTCAAGAACGGCGTAACCATGAGGTAGCTGTAGATGTGCTAGGAAGAACCAATAACGCTATGCTCTGGGTCAACATGTTATCTGGGGACTTTAAGGCGCTTGAGGAAAGAATCGCGCT                   >  1:66600/1‑301`  
                                                                                                                                                                                                                                                                                       `aGAATACTTGGAGGAAGTCGAGGAAGTCGCACGACAGAAAGAAATTGACCGCGCTAAGGCCCGTAAAGAACGTCACGAGGGGCGCTTAGAGGCACGCAGATTCAAACGTCGCAACCGCAAGGCACGTAAAGCACACAAAGCTAAGCGCGAAAGAATGCTTGCTGCGTGGCGATGGGCTGAACGTCAAGAACGGCGTAACCATGAGGTAGCTGTAGATGTACTAGGAAGAACCAATAACGCTATGCTCTGGGTCAACATGTTCTCTGGGGACTTTAAGGCGCTTGATGAACGAATCGCGCTG                  <  1:252889/300‑1`  
                                                                                                                                                                                                                                                                                        `GAATACTTGGAGGAAGTCGAGGAAGTCGCACGACAGAAAGAAATTGACCGCGCTAAGGCCCGTAAAGAACGTCACGAGGGGCGCTTAGAGGCACGCAGATTCAAACGTCGCAACCGCAAGGCACGTAAAGCACACAAAGCTAAGCGCGAAAGAATGCTTGCTGCGTGGCGATGGGCTGAACGTCAAGAACGGCGTAACCATGAGGTAGCTGTAGATGTACTAGGAAGAACCAATAACGCTATGCTCTGGGTCAACATGTTCTCTGGGGACTTTAAGGCGCTTGAGGAACGACTCGCGCTGC                 >  1:567020/1‑301`  
                                                                                                                                                                                                                                                                                        `GAATACTTGGAGGAAGTCGAGGAAGTCGCACGACAGAAAGAAATTGACCGCGCTAAGGCCCGTAAAGAACGTCACGAGGGGCGCTTAGAGGCACGCAGATTCAAACGTCGCAACCGCAAGGCACGTAAAGCACACAAAACTAAGCGCGAAAGAATGCTTGCTGCGTGGCGATGGGCTGAACGTCAAGAACGGCGTAACCATGAGGTAGCTGTAGAGGTACTAGGAAGAACCAATAACGCGATGCTCTGGGTCAACATGTTCTCTGGGGACGTTAAGGCGCTTGAGGAACGAATCtggctct                 >  1:327179/1‑294`  
                                                                                                                                                                                                                                                                                          `atataagaagcagGTCGAGGAAGTCACACGACAGAAAGAAATTGACCGCGATAAGGCCCGTAAAGAACGTCACGAGTGGCGCTGAGAGGCACGCAGATGCAAACGTCGCAACCGCAAGGCACGTAAAGCACACAAAGCTAAGCGCGAAAGAATGCTGGCTGCGTGGCGATGGGCTGAACGTCAAGAACGGCGTAACCATGAGGTAGCTGTAGATGTACTAGGAAGAACCAATAACGCTATGCTCTGGGTCAACATGTTCTCTGGGGACTTTAAGGCGCTTGAGGAACGAATCGCGCTGCAC               <  1:465528/288‑1`  
                                                                                                                                                                                                                                                                                          `ATACTTGGGGGAAGTCGAGGAAGTCGCACGACAGAAAGAAATTGACCGCGCTAAGGCCCGTAAAGAACGTCACGAGGGGCGCTTAGAGGCACGCAGATTCAAACGTCGCAACCGCAAGGCACGTAAAGCACACAAAGCTAAGCGCGAAAGAATGCTTGCTGCGTGGCGATGGGCTGAACGTCAAGAACGGCGTAACCATGAGGTAGCTGTAGATGTACTAGGAAGAACCAATAACGCTATGCTCTGGGTCAACATGTTCTCTGGGGACTTTAAGGCGCTTGAGGAACGAATCGCGCTGCAC               <  1:4907/301‑1`  
                                                                                                                                                                                                                                                                                          `ATACTTGGAGGAAGTCGAGGAAGTCGCACGACAGAAAGAAATTGACCGCGCTAAGGCCCGTAAAGAACGTCACGAGGGGCGCTTAGAGGCACGCAGATTCAAACGTCGCAACCGCAAGGCACGTAAAGCACACAAAGCTAAGCGCGAAAGAATGCTTGCTGCGTGGCGATGGGCTGAACGTCAAGAACGGCGTAACCATGAGGTAGCTGTAGATGTACTAGGAAGAACCAATAACGCTATGCTCTGGGTCAACATGTTCTCTGGGGACTTTAAGGCGCTTGAGGAACGAATCGCGCTGCAC               >  1:300021/1‑301`  
                                                                                                                                                                                                                                                                                          `ATACTTGGAGGAAGTCGAGGAAGTCGCACGACAGAAAGAAATTGACCGCGCTAAGGCCCGTAAAGAACGTCACGAGGGGCGCTTAGAGGCACGCAGATTCAAACGTCGCAACCGCAAGGCACGTAAAGCACACAAAGCTAAGCGCGAAAGAATGCTTGCTGCGTGGCGATGGGCTGAACGTCAAGAACGGCGTAACCATGAGGTAGCTGTAGATGTACTAGGAAGAACCAATAACGCTATGCTCTGGGTCAACATGTTCTCTGGGGACTTTAAGGCGCTTGAGGAACGAATCGCGCTGCAC               <  1:594061/301‑1`  
                                                                                                                                                                                                                                                                                          `ATACTTGGAGGAAGTCGAGGAAGTCGCACGACAGAAAGAAATTGACCGCGCTAAGGCCCGTAAAGAACGTCACGAGGGGCGCTTAGAGGCACGCAGATTCAAACGTCGCAACCGCAAGGCACGTAAAGCACACAAAGCTAAGCGCGAAAGAATGCTTGCTGCGTGGCGATAGGCTGAACGTCAAGAACGGCGTAACCATGAGGTAGCTGTAGATGTACTAGGAAGAACCAATACCGCTATGCTCTGGGTCAACATGTTCTCTGGGGACTTTACGGCGCTTGAGGAACGAATCGCGCTTCAC               >  1:355361/1‑301`  
                                                                                                                                                                                                                                                                                            `ACTTGGAGGAGGTCGAGGAAGTCGAGCGACAGAAAGGAATTGACCGCGGTAAGGCCCGTAAAGAACGTCACGAGGGGCGCTTGGAGGCACGCAGATTCAAACGGCGCAACCGCAAGGCACGTAAAGCACACAAAGCTAAGCGCGAAAGAATGCGTGCTGCGTGGCGATGGGGTGAACGTCAAGAACGGCGTAACCATGAGGTAGCTGTAGATGTACTCGGAAGAACCAATAACGCTATGCTCTGGGTCAACATGTTCTCTGGGGACTTTAAGGCGCTTGAGGAACGAATCGCGCTGCACTG             >  1:605036/1‑301`  
                                                                                                                                                                                                                                                                                            `ACTTGGAGGAAGTCGAGGAAGTCGCACGACAGAAAGAAATTGACCGCGCTAAGGCCCTTAAAGAACGTCACGAGGGGCCCTTAAAGGCACGCAGATTCAAACGTCGAAACCGCAAGGCACGTAAAGCACACAAACCTAAGCGCGAAGAAATGCTTGCTCCGTGGCGATGGGCTGAACGTCAAGAACGGCGTAACCATGAGGAAGCTGTAAATGAACTAGGAAGAACCAAAAACCCCATACTCTGGTTCAACATGTTCTCTGGGGACTTTAAGGCGCTGCAGGAACGAATCGCGCTGCACTG             >  1:315973/1‑301`  
                                                                                                                                                                                                                                                                                             `tgTGGAGGAAGTCGAGGAAGGCGAACGACAGAAAGAAATTGACGGAGCGAAGGCCCGAAAAGGACGTCAGGAAGGGCGCTTAGAGGCACGCAGATTCAAACGTCGCAACCGCAAGGCACGGAAAGCACACAAATCTAAGCGCGAAAGGATGCTTGCTGCGTGGCGATGGGCTGAACGTCAAGAACGGCGTAACCATGAGGTAGCTGTAGATGTACTAGGAAGAACCAATAACGCTATGCTCTGGGTCAACGTGTTCTCTGGGGACTTTAAGGCGCTTGAGGAACGAATCGCGCTGCACTGG            <  1:421781/299‑1`  
                                                                                                                                                                                                                                                                                             `CTTGGAGGAAGTCGAGGAAGTCGCACGACAGAAAGAAATTGACCGCGCTAAGGCCCGTAAAGAACGTCACGAGGGGCGCTTAGAGGCACGCAGATTCAAACGTCGCAACCGCAAGGCACGTAAAGCACACAAAGCTACGCGCGAAAGAATGCTTGCTGCGTGGCGATGGGCTGAACGTCAAGAACGGCGTAACCATGAGGTAGCTGTAGATGTACTAGGGAGAACCAATAACGCTATGCTCTGGGTCAACATGTTCTCTGGGGACTTTAAGGCGCTTGAGGAACGAATCGCGCTGCACTGG            >  1:294398/1‑301`  
                                                                                                                                                                                                                                                                                             `CTTGGAGGAAGTCGAGGAAGTCGCACGACAGAAAGAAATTGACCGCGCTAAGGCCCGTAAAGAACGTCACGAGGGGCGCTTAGAGGCACGCAGATTCAAACGTCGCAACCGCAAGGCACGTAAAGCACACAAAGCTAAGCGCGAAAGAATGCTTGCTGCGTGGCGATGGGCTGAACGTCAAGAACGGCGTAACCATGAGGTAGCTGTAGATGTACTAGGAAGAACCAATAACGCTATGCTCTGGGTCAACATGTTCTCTGGGGACTTTAAGGCGCTTGAGGAACGAATCGCGCTGCACTGG            >  1:570027/1‑301`  
                                                                                                                                                                                                                                                                                               `gGGAGGAAGTCGAGGAAGTCGCACGACAGAAAGAAATTGACCGCGCTAAGGCCCGTAAAGAACGTCACGAGGGGCGCTTAGAGGCACGCAGATTCAAACGTCGCAACCGCAAGGCACGTAAAGCACACAAAGCTAAGCGCGAAAGAATGCTTGCTGCGTGGCGATGGGCTGAACGTCAAGAACGGCGTAACCATGAGGTAGCTGTAGATGTACTAGGAAGAACCAATAACGCTATGCTCTGGGTCAACATGTTCTCTGGGGACTTTAAGGCGCTTGAGGAACGAATCGCGCTGCACTGGCG          <  1:345636/300‑1`  
                                                                                                                                                                                                                                                                                               `aGGAGGAAGTCGAGGAAGTCGCACGACAGAAAGAAATTGACCGCGCTAAGGCCCGGAAAGAACGTCACGAGGGGCGCTTAGAGGCACGCAGATTCAAACGTCGCAACCGCAAGGCACGTAAAGCACACAAAGCTAAGCGCGAAAGAATGCTGTCTGCGTGGCGATGGGCTGAACTTCAAGAACGGCGTAACCATGAGGTAGCTGTAGATGTACTAGGAAGAACCAATAACGCTATGCTCTGGGTCAACATGTTCTCTGGGGACTTTAATGCGCTTGAGGAACGAATCGCGCTGCACTGGCG          <  1:643654/300‑1`  
                                                                                                                                                                                                                                                                                                `GGAGGAAGTCGAGGAAGTCGCACGACAGAAAGAAATTGACCGCGCTAAGGCCCGTAAAGAACGTCACGAGGGGCGCTTAGAGGCACGCAGATTCAAACGTCGCAACCGCAAGGCACGTAAAGCACACAAAGCTAAGCGCGAAAGAATGCTTGCTGCGTGGCGATGGGCTGAACGTCAAGAACGGCGTAACCATGAGGTAGCTGTAGATGTACTAGGAAGAACCAATAACGCTATGCTCTGGGTCAACATGTTCTCTGGGGACTTTAAGGCGCTTGAGGAACGAATCGCGCTGCACTGGCGT         >  1:271446/1‑301`  
                                                                                                                                                                                                                                                                                                 `GAGGAAGTCGAGGAAGTCGCACGACAGAAAGAAATTGACCGCGCTAAGGCCCGTAAAGAACGTCACGAGGGGCGCTTAGAGGCACGCAGATTCAAACGTCGCAACCGCAAGGCACGTAAAGCACACAAAGCTAAGCGCGAAAGAATGCTTGCTGCGTGGCGATGGGCTGAACGTCAAGAACGGCGTAACCATGAGGTAGCTGTAGATTTACTAGGAAGAACCAATAACACTATTATCTGGGGCAACATGTTCTCTGTGGACTTTAAGGCGCCTGAGGAACGAATCGCACTGCACTTGCTTA        >  1:495108/1‑301`  
                                                                                                                                                                                                                                                                                                 `GAGGAAGTCGAGGAAGTCGCACGACAGAAAGAAATTGACCGCGCTAAGGCCCGTAAAGAACGTCACGAGGGGCGCTTAGAGGCACGCAGATTCAAACGTCGCAACCGCAAGGCACGTAAAGCACACAAAGCTAAGCGCGAAAGAATGCTTGCTGCGTGGCGATGGGCTGAACGTCAAGAACGGCGTAACCATGAGGTAGCTGTAGATGTACTAGGAAGAACCAATAACGCTATGCTCTGGGTCAACATGTTCTCTGGGGACTTTAAGGCGCTTGAGGAACGAATCTCGCTGCACTGGCGTA        >  1:504695/1‑301`  
                                                                                                                                                                                                                                                                                                    `GAAGTCGAGGAAGTCGCACGACAGAAAGAAATTGACCGCGCTAAGGCCCGTAAAGAACGTCACGA‑GGGCGCTTAGAGGCACGCAGATTCAAACGTCGCAACCGCAAGGCACGTAAAGCACACAAAGCTAAGCGCGAAAGAATGCTTGCTGCGTGGCGATGGGCTGAACGTCAAGAACGGCGTAACCATGAGGTAGCTGTAGATGTACTAGGAAGAACCAATAACGCTATGCTCTGGGTCAACATGTTCTCTGGGGACTTTAAGGCGCTTGAGGAACGAATCGCGCTGCACTGGCGTAATGC    <  1:101714/301‑1`  
                                                                                                                                                                                                                                                                                                    `GAAGTCGAGGAAGTCGCAAGACAGAAAGAAAGTGACCGCGCTAAGGCCCGTAAAGAACGTCACGAGGGGCGCTTAGAGGCACGCAGATTCAAACGTCGCAACCGCAAGGCACGTAAAGCACACAAAGCTAAGCGCGAAAGAATGCTTGTTGCGTGGCGATGGGCTGAACGTCAAGAACGGCGTAACCATGAGGTAGCTGTAGATGTACTAGGAAGAACCAATACCGCTATGCTCTGGGTCAACATGTTCTCTGGGGTCTTTAAGGCGCTTGAGGAACGAATCGCGCTGCACTGGCGTAATG     <  1:95301/301‑1`  
                                                                                                                                                                                                                                                                                                     `acttgagGGAAGTCGCACGACAGAAAGAACTTCACCTCGCTAAGTCCCGTAAATAACGTCACGAGGGGCGCTTCGAGGCACGCAGATTCAAACGTCGCAACCGCAAGGCACGTAAAGCACACCAAGCTAAGCGCGAAAGAATGCTTGCTGCGTGGCGATGGGCTGAACGTCAAGAACGGCGTAACCATGAGGTAGCTGTAGATGTACTAGGAAGAACCAATAACGCTATGCTCTGGTTCAACATGTTCTCTGGGGACTTTAAGGCGCTTGAGGAACGAATCGCGCTGCACTGGCGTAATGC    <  1:55375/294‑1`  
                                                                                                                                                                                                                                                                                                     `AAGTCGAGGAAGTCGCACGACAGAAAGAAATTGACCGCGCTAAGGCACGTAAAGAACGTCACGAGGGGCGCTTAGAGGCACGCAGATTCAAACGTCGCAACCGCAAGGCACGTAAAGCACACAAAGCTAAGCGCGAAAGAATGCTTGCTGCGTGGCGATGGGCTGATCGTCAAGAACGGCGTAACCATGAGGTAGCTGTAGATGTACTAGGAAGAACCAATAACGCTATGCTCTGGGTCAACATGTTCTCTGGGGACTTTAAGGCGCTTGAAGAACGAATCGCGCTGCACTGGCGTAATGC    <  1:209559/301‑1`  
                                                                                                                                                                                                                                                                                                     `AAGTCGAGGAAGTCGAACGACAGAAAGAAATTGACCGCGCTAAGGCCCGTAAAGAACGTCACGAGGGGCGCTTAGAGGCACGCAGATTCAAACGTCGCAACCGCAAGGCACGTAAAGCACACAAAGCTAAGCGCGAAAGAATGCTTGCTGCGTGGCGATGGGCTGAACGTCAAGAACGGCGTAACCATGAGGTAGCTGTAGATGTACTAGGAAGAACCAATAACGCTATGCTCTGGGTCAACATGTTCTCTGGGGACTTTAAGGCGCTTGAGGAACGAATCGCGCTGCACTGGCGTAATGC    <  1:263705/301‑1`  
                                                                                                                                                                                                                                                                                                     `AAGACGAGGAAGTCGAACGACAGAAAGAAATGGACCGAGGTAAGGCCAGTAAAGAACGGCACGAGGGGCGCTTAGAAGAACGCAGATTCAAACGTCGCAACCGCAAGGAACGTAAAGCACACAAAGCTAAGCGCGAAAGAATGCTTGCTGCGTGGCGATGGGCTGAACGTCAAGAACGGCGTAACCATGAGGTAGCTGTAGATGTACGAGGAAGAACCAATAACGCTATGCTCTGGGTCAACATGTTCTCTTGGGACTTTAAGGCGCTTGAGGAACGAATCGCGCTGCACTGGCGTAATGC    <  1:65126/301‑1`  
                                                                                                                                                                                                                                                                                                       `GTCGAGGAAGTCGCACGACAGAAAGAAATTGACCGCGCTAAGGCCCGTAAAGAACGTCACGAGGGGCGCTTAGAGGCACGCAGATTCAAACGTCGCAACCGCAAGGCACGTAAAGCACACAAAGCTAAGCGCGAAAGGATGCTTGCTGCGTGGCGATGGGCTGAACGTCAAGAACGGCGTAACCATGAGGTAGCTGTAGATGTACTAGGAAGAACCAATAACGCTATGCTCTGGGTCAACATGTTCTCTGGGGACTTTAAGGCGCTTGAGGAACGAATCGCGCTGCACTGGCGTAATGCTt  >  1:285545/1‑300`  
                                                                                                                                                                                                                                                                                                       `GTCGAGGAAGTCGCACGACAGAAAGAAATTGACCGCGCTAAGGCCCGTAAAGAACGTCACGAGGGGCGCTTAGAGGCACGCAGATTCAAACGTCGCAACCGCAAGGCACGTAAAGCACACAAAGCTAAGCGCGAAAGAATGCTTGCTGCGTGGCGATGGGCTGAACGTCAAGAACGGCGTAACCATGAGGTAGCTGTAGATGTACTAGGAAGAACCAATAACGCTATGCTCTGGGTCAACATGTTCTCTGGGGACTTTAAGGCGCTTGAGGAACGAATCGCGCTGCACTctgtctcttata  >  1:344796/1‑289`  

                                                                                                                                                                                                                                                                                                                                                                                                                                                                                                                                                                                                                      
`CGTTTTCGACCACGCTTACGAAATGCTGAAAGAAAACATCCGTTATGATGACATCCGTGACACTGATGACCTGCACGATGCTATTCACATGGCTGCCGATAATGCAGTTCCGCACTACTACGCTGACATCTTTAGCGTAATGGCAAGTGAGGGCATTGACCTTGAGTTCGAAGACTCTGGTCTGATGCCTGACACCAAGGACGTAATCCGCATCCTGCAAGCGCGTATCTATGAGCAATTAACGATTGACCTCTGGGAAGACGCAGAAGACTTGCTCAATGAATACTTGGAGGAAGTCGAGGA‑‑‑‑‑‑‑‑‑‑‑‑‑‑‑‑‑‑‑‑‑‑‑‑‑‑‑‑‑‑‑‑‑‑‑‑‑‑‑‑‑‑‑‑‑‑‑‑‑‑‑‑‑‑‑‑‑‑‑‑‑‑‑‑‑‑‑‑‑‑‑‑‑‑‑‑‑‑‑‑‑‑‑‑‑‑‑‑‑‑‑‑‑‑‑‑‑‑‑‑‑‑‑‑‑‑‑‑‑‑‑‑‑‑‑‑‑‑‑‑‑‑‑‑‑‑‑‑‑‑‑‑‑‑‑‑‑‑‑‑‑‑‑‑‑‑‑‑‑‑‑‑‑‑‑‑‑‑‑‑‑‑‑‑‑‑‑‑‑‑‑‑‑‑‑‑‑‑‑‑‑‑‑‑‑‑‑‑‑‑‑‑‑‑‑‑‑‑‑‑‑‑‑‑‑‑‑‑‑‑‑‑‑‑‑‑‑‑‑‑‑‑‑‑‑‑‑‑‑‑‑‑‑‑‑‑‑‑‑‑‑‑‑‑‑‑‑‑‑‑‑‑‑‑‑‑‑‑‑‑‑‑‑‑‑‑‑‑‑‑‑‑‑‑‑‑‑‑‑‑‑‑‑‑‑‑‑‑‑‑‑‑‑  >  NC_001604.1/954‑1256`  
`‑‑‑‑‑‑‑‑‑‑‑‑‑‑‑‑‑‑‑‑‑‑‑‑‑‑‑‑‑‑‑‑‑‑‑‑‑‑‑‑‑‑‑‑‑‑‑‑‑‑‑‑‑‑‑‑‑‑‑‑‑‑‑‑‑‑‑‑‑‑‑‑‑‑‑‑‑‑‑‑‑‑‑‑‑‑‑‑‑‑‑‑‑‑‑‑‑‑‑‑‑‑‑‑‑‑‑‑‑‑‑‑‑‑‑‑‑‑‑‑‑‑‑‑‑‑‑‑‑‑‑‑‑‑‑‑‑‑‑‑‑‑‑‑‑‑‑‑‑‑‑‑‑‑‑‑‑‑‑‑‑‑‑‑‑‑‑‑‑‑‑‑‑‑‑‑‑‑‑‑‑‑‑‑‑‑‑‑‑‑‑‑‑‑‑‑‑‑‑‑‑‑‑‑‑‑‑‑‑‑‑‑‑‑‑‑‑‑‑‑‑‑‑‑‑‑‑‑‑‑‑‑‑‑‑‑‑‑‑‑‑‑‑‑‑‑‑‑‑‑‑‑‑‑‑‑‑‑‑‑‑‑‑‑‑‑‑‑‑‑‑‑‑‑‑‑‑‑‑‑‑‑‑‑‑‑‑‑‑‑aggaagtcgaggaAGTCGCACGACAGAAAGAAATTGACCGCGCTAAGGCCCGTAAAGAACGTCACGAGGGGCGCTTAGAGGCACGCAGATTCAAACGTCGCAACCGCAAGGCACGTAAAGCACACAAAGCTAAGCGCGAAAGAATGCTTGCTGCGTGGCGATGGGCTGAACGTCAAGAACGGCGTAACCATGAGGTAGCTGTAGATGTACTAGGAAGAACCAATAACGCTATGCTCTGGGTCAACATGTTCTCTGGGGACTTTAAGGCGCTTGAGGAACGAATCGCGCTGCACTGGCGTAATGCTG  >  NC_001604.1/2737‑3029`

  

Alignment Legend

Aligned base mismatch/match (shaded by quality score): `ATCG/ATCG` `< 3 ≤` `ATCG/ATCG` `< 9 ≤` `ATCG/ATCG` `< 14 ≤` `ATCG/ATCG` `< 31 ≤` `ATCG/ATCG` `< 38 ≤` `ATCG/ATCG`

Unaligned base: `atcg`    Masked matching base: `atcg`    Alignment gap: `‑`    Deleted base: `‑`

Reads not counted as support for junction

read\_name Not counted due to insufficient overlap past the breakpoint.

read\_name Not counted due to not crossing MOB target site duplication.
